# Within-sample co-methylation patterns in normal tissues

**DOI:** 10.1186/s13040-019-0198-8

**Published:** 2019-05-09

**Authors:** Lillian Sun, Shuying Sun

**Affiliations:** 1 Stanford University, Stanford, CA USA; 2 Department of Mathematics, Texas State University, San Marcos, TX USA

**Keywords:** Within-sample co-methylation, Normal tissue, Statistical analysis

## Abstract

**Background:**

DNA methylation is an epigenetic event that may regulate gene expression. Because of this regulation role, aberrant DNA methylation is often associated with many diseases. Within-sample DNA co-methylation is the similarity of methylation in nearby cytosine sites of a chromosome. It is important to study co-methylation patterns. However, it is not well studied yet, and it is unclear to us what co-methylation patterns normal DNA samples have. Are the co-methylation patterns of the same tissue across several samples different? Are the co-methylation patterns of various tissues of the same sample different? To answer these questions, we conduct analyses using two sets of data: 3-sample-1-tissue (3S1T) and 1-sample-8-tissue (1S8T).

**Results:**

To study the co-methylation patterns of the two datasets, 3S1T and 1S8T, we investigate the following questions: How often does one methylation state change to other methylation states and how is this change associated with chromosome distance? Based on the 3S1T data, we find there is not significant co-methylation difference among the same spleen tissues of three different samples. However, the analysis results of 1S8T data show that there were significant differences among eight tissues of one sample. For both 3S1T and 1S8T data, we find that the no/low methylation state A and high/full methylation state D tend to remain the same along a chromosome region. We also find that the low/partial methylation state B and partial/high methylation state C tend to change to higher methylation states along a chromosome. Finally, we find that lengths of most co-methylation regions are very short with only a few hundred base pairs. In fact, only a small proportion of methylated regions are longer than 1000 base pairs.

**Conclusions:**

In this paper, we have addressed a few questions regarding within-sample co-methylation patterns in normal tissues. Our statistical analysis results and answers may help researchers to better understand the biological process of DNA methylation. This may pave the way to develop better analysis methods for future methylation research.

**Electronic supplementary material:**

The online version of this article (10.1186/s13040-019-0198-8) contains supplementary material, which is available to authorized users.

## Introduction

Epigenetics is generally understood as the study of heritable changes that are related to gene functions and cannot be explained by changes in DNA sequences. These changes are frequently termed epigenetic events or marks. One main type of epigenetic event is DNA methylation, which is the addition of a methyl group to a 5′ cytosine base [1]. DNA methylation plays an important role in transcription and thus affects gene expression [1, 2]. It also plays a significant role in genomic imprinting, X-chromosome inactivation, and suppression of repetitive element transcription and transposition [3–7]. Therefore, it is important to study different methylation patterns in both normal samples and the samples of complex diseases such as cancers.

In a human genome, DNA methylation often occurs at CpG or CG sites. A CG site is a cytosine base followed by a guanine base in DNA sequences. Because DNA methylation predominantly occurs at CG sites, it is important to study DNA methylation at or near CpG islands, which are regions with more cytosine or CG sites. CpG islands are defined as regions of the DNA sequence > 200 bp long with an average CG content > 50% and an observed-to-expected ratio of CG sites > 0.6 [8]. These islands are important regulatory elements in the genome, and contain the most variation in DNA methylation across different tissues. Additionally, methylation of CpG islands in promoter regions can be associated with long-term silencing of gene expression [2, 9].

Researchers can study segments of methylation over a stretch of neighboring CG sites in the same chromosome region. This specific DNA methylation pattern is called co-methylation [10]. It is also called within-sample (WS) co-methylation. The prefix “co-” in the “WS co-methylation” means local CG sites of one short chromosome region methylate or not methylate together in a single sample. That is, we study how neighboring CG sites are similarly methylated or unmethylated within a short chromosome region of one sample. It has also been observed that the “correlation between methylated cytosines decays as a function of genomic distance between methylated loci”. In fact, in Arabidopsis this correlation is observed for distances up to 5000 nucleotides [11]. For human samples, Eckhardt et al. first report that this correlation or co-methylation is over short distances (≤ 1000 base pairs) and it deteriorates rapidly for distances > 2000 base pairs [12]. However, this study is not conducted for the whole genome. In this paper, we will investigate in detail how long this co-methylation pattern can be in the human genome of normal samples using whole genome bisulfite sequencing data.

Before we introduce our study further, we emphasize that, in other research papers [13–20]*,* co-methylation may mean “between-sample (BS) co-methylation”. The prefix “co-” in the “BS co-methylation” means that genes at different regions, especially different chromosomes, methylate together across multiple samples. For example, “BS co-methylation” may mean that two or more genes (e.g., genes 1 and 2) on different chromosomes are hypermethylated together and their functions are somehow related through a co-methylation (or co-expression) network/module [15, 19]. This co-methylation (or “BS co-methylation”) is similar to the concept of “co-expression” of genes. The above explanations and definitions of WS co-methylation and BS co-methylation is consistent with the ones defined in Peter Francis Hickey’s thesis [21], which has a thorough review of different co-methylation definitions. It is worth noting that although “WS co-methylation” is about the methylation pattern within a single sample, it is important to study “WS co-methylation” across multiple samples and this is the research focus of this paper. To simplify our writing, in this paper we often use “co-methylation” for “WS co-methylation*”.*

It is important to study within-sample co-methylation as it explains how DNA methylation is instituted in each genomic region in a chromosome. Deep understanding of within-sample co-methylation patterns can help researchers improve DNA methylation assays and statistical analyses of DNA methylation. Thus, we can better understand other genetic and epigenetic events or patterns. Although it is important to study DNA co-methylation, this specific methylation pattern has not been well studied yet. For example, it is unclear to us what co-methylation patterns normal DNA samples have. In this paper, we will conduct analyses to answer the following two questions regarding co-methylation patterns in normal DNA. First, are the co-methylation patterns of the same tissue across several samples different? Second, are the co-methylation patterns of various tissues of the same sample different? In order to answer these questions, we conduct analyses using two sets of data: one is 3-sample-1-tissue (3S1T), another one is 1-sample-8-tissue (1S8T). To study the co-methylation patterns of these two datasets, we specifically investigate how often one methylation state change to other methylation states and how is this change associated with chromosome distance. In the following sections, we first introduce the data we use. We then explain the two analysis methods we will use to answer the above questions. Finally, we will show our comprehensive analysis results.

## Methods

### Data

To compare co-methylation patterns among different samples, we use a dataset of three distinct samples. The samples are referred to as STL001, STL002, and STL003 and each sample has whole genome bisulfite sequencing (WGBS) data from the spleen tissue. These datasets are obtained from the Roadmap Epigenomics Project [22]. We refer to this dataset as 3-sample-1-tissue, or 3S1T. To compare co-methylation patterns among different tissues of the same sample, we use a dataset of eight distinct tissues of the sample STL001. The eight tissues are respectively referred to as bladder, gastric, lung, psoas, sigmoid colon, small bowel, spleen, and thymus. This dataset is obtained from Roadmap Epigenomics Project [22]. We refer to this dataset as 1-sample-8-tissue, or 1S8T. For both 3S1T and 1S8T data, the raw WGBS reads are preprocessed and aligned using BRAT-bw [23], a publicly available software package, and the human genome hg19 is used as the reference genome. Because our focus is within-sample co-methylation for nearby sites or regions, not between-sample co-methylation in a whole genome, using one chromosome is sufficient to address the questions of interest. Therefore, we only focus on chromosome 1, as it is the longest chromosome. In fact, we use methylation ratios from all CG sites in chromosome 1 of each dataset.

Each methylation sequencing dataset includes four metrics: chromosome, position, sequencing coverage, and MC ratio, see Table 1. At a specific CG site, the “MC ratio” is defined as the number of reads with methylated cytosines divided by the total number of reads covering that CG site. It is similar to the “beta value” that is commonly used for the methylation signals of Illumina array probes. “MC ratio” ranges from 0 to 1 and is the proportion of sequenced reads of a CG site that are methylated. 0 indicates no methylation and 1 indicates full methylation. We then add a fifth metric—the methylation state of each CG site—to all datasets. “Methylation state” refers to each of our divisions of the MC ratio [0,1] into four intervals of methylation. These methylation states are A, B, C, and D. “A” corresponds to no/low MC ratios of [0, 0.25). “B” corresponds to low/partial MC ratios of [0.25, 0.5). “C” corresponds to partial/high MC ratios of [0.5, 0.75). “D” corresponds to high/full MC ratios of [0.75, 1]. CG sites with a sequencing coverage of less than 3 (i.e., <3X) are labeled “NA”. Note, as for the sequencing coverage, for all datasets (samples/tissues), around 95% of CG sites have at least 1X coverage. Only two samples/tissues have 89 and 91% of CG sites with ≥3X coverage. All other samples/tissues have about 95% of CG sites with ≥3X coverage. Lastly, we calculate the distance between consecutive CG sites and add these values in the final column of our data (Table 1). “Consecutive CG sites” mean two CG sites on the same chromosome and at the same DNA strand, that is, there is no other CG site between them. The first and second CG sites are determined based on the reference genome coordinates on the positive or forward strand. In this paper, we only use the positive or forward strand of the DNA.

**Table 1:** Sample section of data

Chr	Position	Sequence Coverage	MC Ratio	Methylation State	Distance
chr1	434,314	20	.3	B	12
chr1	434,326	19	1	D	3
chr1	434,329	17	1	D	14
chr1	434,343	15	0.20	A	17
chr1	434,360	20	0.8	D	45
chr1	434,405	21	0.67	C	31
chr1	434,436	0	NA	NA	10

### Two analyses

We prepare the original data as described above, labeling the methylation state of each CG site and calculating distance between consecutive CG sites, so that we may use them in our two analyses. First, we investigate how frequently methylation states change from one CG site to another along a chromosome. Second, we investigate if this change is related to chromosome distance.

**Fig. 1:**
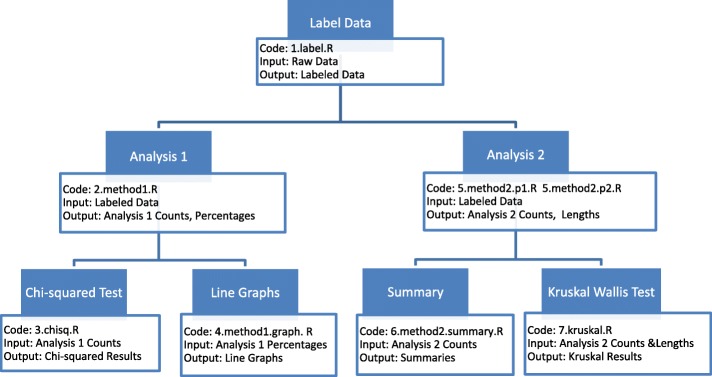
Workflow of Analysis Methods. In each step, the R code, input, and output files are listed. R code files listed in this figure are provided in the Additional file 1

We would like to investigate patterns beyond the scope of single sample or tissue. Do the co-methylation patterns vary across different samples or different tissues? We apply our analyses to the different samples and tissues in two datasets, 3S1T and 1S8T. We compare co-methylation patterns across different samples in 3S1T and compare co-methylation across different tissues in 1S8T to answer these questions. The workflow of our analyses is shown in Fig. 1. Next, we explain in detail each analysis method.

#### Analysis 1

In our first analysis, we analyze how methylation patterns change from one CG site to another. Since each CG site has a methylation state assigned to it according to its methylation level, we pair together consecutive CG sites along the genome to form a “methylation state pair”—the two states corresponding to a pair of CG sites. This allows us to observe how methylation can change along a chromosome region. The first state indicates the initial methylation level, while the second state indicates the terminal methylation level. Does methylation stay the same (e.g., AA, BB, CC, DD), relatively similar (e.g., AB, CD), or change drastically (e.g., AD, DA)? Do certain methylation states tend to change to other methylation states?

For each sample, we also calculate the frequency of the distinct state pairs (e.g., AA, AB, AC, AD). This allows us to compare the co-methylation patterns across different samples or tissues (see Chi-squared test below). We learned from our previous research that co-methylation patterns are related to the physical distance between two CG sites in a state pair. Thus, we calculate the frequency of each type of methylation state-pair at increasing intervals of a short distance of 50 bases, i.e., [0, 50), [50, 100), [100, 150), …, [400, 450), [450, 500), to [500, Inf). We then graph the frequencies of different samples or tissues with the same methylation state pair on the same graph to visually observe differences. We also utilize the chi-squared test to determine whether samples or tissues are significantly different. We first test all samples or tissues at once. If there is a significant difference, we further analyze the data by pairwise comparison to determine which combination of samples may be the cause of the difference. If the *p*-values are extremely small, we may use or combine approaches below to produce better analysis results.

- We calculate the contribution of each methylation state pair and each sample to a Chi-squared statistic. This allows us to determine which sample and which methylation state pair is the most significantly different from the others. We display this information in bar graphs for visualization.
- We divide our methylation state-pair count data (i.e., Chi-squared input) by factors of n (*n* = 10, 100, 1000) to account for large-count issues.

#### Analysis 2

In our second analysis, we investigate how long methylation levels stay the same in a chromosome by analyzing the length of similarly methylated regions. We begin by grouping together consecutive CG sites that have the same methylation state (e.g., AAAA, BBBB, CCCC, or DDDD). These groups are called similarly methylated regions (SMRs). We then calculate the number of CG sites in each group (count) and the number of base pairs the group stretches across (region-length or length). These two values are used as metrics that we use to compare different samples or tissues.

Once we calculate counts and lengths, we summarize the counts and lengths of the four different SMRs: A, B, C, and D. We summarize SMRs with a count of at least two CG sites. We then determine whether SMRs from different samples or tissues with the same methylation level (i.e., AAAA in Bladder and AAAAA in Thymus) have significantly different distributions of lengths or of counts. We begin to analyze SMR count/length summaries across different samples or tissues. These summaries provide a preliminary assessment of whether SMR lengths and counts from different tissues or samples may differ. We then use the Kruskal Wallis test to confirm any significant differences, first comparing all samples or tissues and then conducting pairwise comparisons, if necessary.

## Results

### 3S1T data analysis results

First, we show the results of analyzing if different samples of the same tissue have different co-methylation patterns.

#### Analysis 1 result of 3S1T data

We have labeled and paired consecutive CG sites in the data to form methylation state pairs. Table 2 displays the number of times each methylation state pair occurs and the relative frequency of methylation state pair. With this summary, we can answer the questions about how methylation changes from one methylation state to another.

**Table 2:** Methylation state change of the 3S1T data

	Count				Percentage			
	A	B	C	D	A	B	C	D
sample 1 - STL001								
A	159,513	12,082	7505	16,928	81.37	6.16	3.83	8.64
B	12,129	13,298	14,876	30,868	17.04	18.69	20.9	43.37
C	7398	15,037	41,996	119,380	4.03	8.18	22.85	64.95
D	17,048	30,890	119,492	1,425,036	1.07	1.94	7.5	89.49
sample 2 - STL002								
A	170,787	13,663	9638	18,661	80.28	6.42	4.53	8.77
B	13,745	17,814	21,403	35,313	15.57	20.18	24.25	40
C	9592	21,348	63,698	151,667	3.89	8.67	25.86	61.58
D	18,723	35,534	151,708	1,308,261	1.24	2.35	10.02	86.4
sample 3 - STL003								
A	209,282	13,179	8953	17,408	84.11	5.3	3.6	7
B	13,282	20,626	22,691	34,669	14.55	22.6	24.86	37.99
C	9104	22,403	71,035	154,423	3.54	8.72	27.64	60.1
D	17,244	35,103	154,381	1,342,781	1.11	2.27	9.96	86.66

The “A”, “B”, “C”, and “D” **rows** indicate the first methylation state of the CG pair. The “A”, “B”, “C”, and “D” **columns** indicate the second methylation state of the pair. The “Count” columns display the number of occurrences of each methylation state pair. The “Percentage” columns display the count of each methylation state pair divided by the row sum of the counts. For example, for sample 1 (STL001), in the “AA” cell, the count is 159,513 (the first number in the table), and the percentage is 81.37%. This means that among all the total 196,028 methylation state of A, 159,513 or 81.37% of the times that the next methylation state is still A

For the STL001 spleen data in Table 2, methylation state pair DD occurs the most (89.49%), followed closely by the methylation state pair AA (81.37%). This means that no/low (A) or high/full (D) methylation states in the STL001 spleen sample tends to stay within the same methylation state. For example, when looking at just row A, across columns A, B, C, and D, the no/low methylation state A tends to remain the same methylation state, no/low (A). On the other hand, low/partial methylation state B and partial/high methylation state C tends to change to higher methylation states: C and D. For high/full methylation state D, the methylation tends to remain high/full (D). There is not a large percentage of methylation state change from D to A (only about 1%). The STL002 and STL003 spleen samples follow similar patterns with some variation in the actual values.

#### Comparing co-methylation patterns among 3 samples of the spleen tissue (i.e., 3S1T data)

**Fig. 2:**
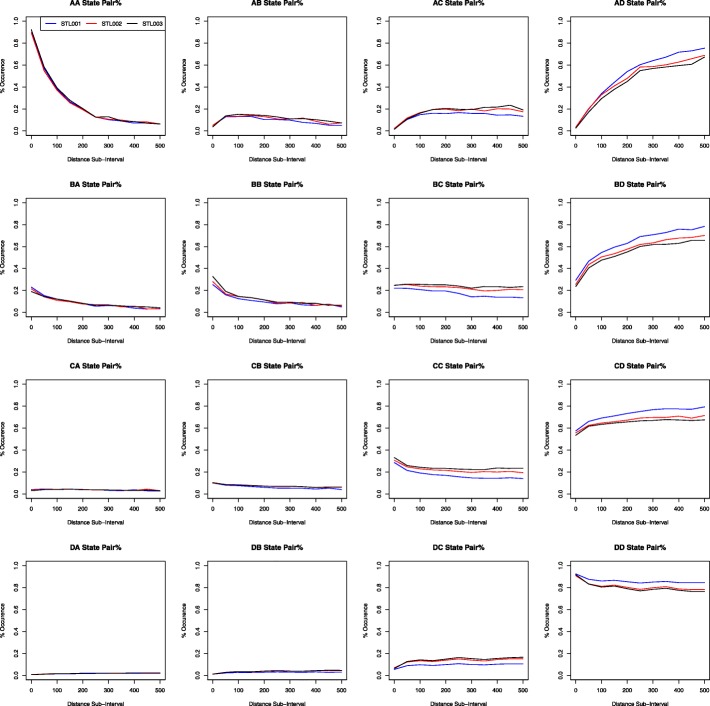
Comparing co-methylation patterns among three spleen samples (for 3S1T data). These graphs are created by plotting the percent occurrence for each distance sub-interval incremented by 50: [0, 50), [50, 100), [100, 150), and so on to [500, Inf). The x-axis indicates distance between CG sites. For example, a distance sub-interval of [50, 100) means that all paired CG sites are within 50 to 100 base pairs of each other. The y-axis indicates the percent occurrence of the methylation state pair as designated by the graph title. The solid red line, the dashed blue line, and the dotted black line indicate the trend of the STL001, STL002, and STL003 spleen sample respectively

Since we have observed some variation in the co-methylation patterns among 3 samples of spleen tissue, we want to determine whether these differences are statistically significant. Firstly, we use a visual depiction to ballpark whether the differences are significant, see Fig. 2. Plots in Fig. 2 do not display an obvious difference in change patterns among the three spleen datasets. The three lines depicting each sample do not vary from each other greatly, except that there is a bit of variation between samples in the higher methylation states, e.g., AD, BD, CD, and DD state pairs.

Next, we use chi-squared tests to study whether there are statistically significant differences for the co-methylation patterns of three samples. The null (H_0_) and alternative (H_a_) hypotheses of the chi-squared tests are listed below. H_0_: There is no difference for the co-methylation patterns among the spleen tissue of three samples. That is, the methylation state change patterns from each methylation state (e.g., A) to all states (A, B, C, D) are the same among the spleen tissues of three samples. H_a_: There are differences for the co-methylation patterns among the spleen tissue of three samples. We use our original count data in Table 2 as the input for each sample to conduct the chi-squared test. We first test all three samples for differences. If the results for all three are significant, we will determine which subset of samples are different: we further test pairs of samples.

**Fig. 3:**
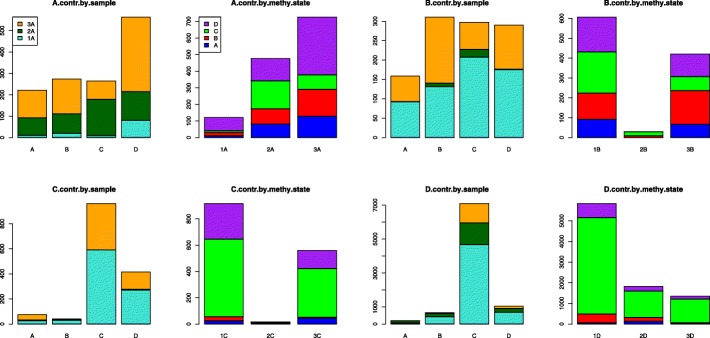
Comparing chi-squared test contributions by sample and by methylation state (for 3S1T data). The title indicates the co-methylation pattern compared by the chi-squared test. The x-axis reveals the second methylation state of the pair. For example, if the graph title contains “A”, then bar “B” displays the contribution of the specific state pair “AB” to the chi-squared statistic. The x-axis can also indicate sample number (i.e., “1C”, “2C”, “3C”) with 1, 2, 3 corresponding to STL001, STL002, and STL003 respectively. The y-axis displays the chi-squared contribution value

The chi-squared test results for all three spleen samples of 3S1T dataset are shown in Table 3. The *p*-values are very small, almost zero. As indicated above, we then conduct pair-wise comparisons between each two of the three samples and we still obtain extremely small *p*-values (data not shown), which shows that three spleen samples are statistically different. However, we suspect that there may be some outlying co-methylation patterns that causes our p-value to be extremely small. Therefore, we compare the chi-squared contributions of each of the co-methylation patterns, see Table 4 and Fig. 3. Table 4 shows that in each of the three samples, some methylation state pairs contribute more than others. For example, the DC methylation-state-pair contributions of spleen samples 1, 2, and 3 are significantly higher than other methylation state pairs. They are 4666.12, 1279.32, 1148.63, as shown in the fourth column of the bottom panel of Table 4.

**Table 3:** Chi-squared test results of 3S1T data

	A	B	C	D
*p*-value	1.06E-282	4.98E-225	1.80E-319	0
Chi-square	1323.14	1056.65	1492.95	9013.65
Degree of freedom	6	6	6	6

Each column indicates the result of a separate chi-squared test (i.e., p-value, statistics, and degree of freedom). The labels “A”, “B”, “C”, and “D” designate which co-methylation patterns/states are compared in the test. For example, “A” means that the chi-squared test compares the percentage occurrence of all state pairs beginning with state “A” (AA, AB, AC, and AD). The input used for the chi-squared test of methylation state “A” is the counts in the row A of samples 1, 2, 3 in Table 2 (that is, the three rows starting with these numbers 159,513, 170,787, and 209,282)

**Table 4:** Contributions of each methylation state pair to chi-squared values (for 3S1T data)

	A	B	C	D	Total
1A	11.07	19.76	9.66	80.79	
2A	81.88	90.94	169.23	133.90	1323.14
3A	128.16	162.92	85.94	348.87	
1B	92.44	131.37	207.56	175.17	
2B	0.13	8.90	19.73	1.08	1056.65
3B	66.29	170.44	69.79	113.76	
1C	24.94	30.29	590.40	271.24	
2C	6.05	3.55	1.87	4.80	1492.95
3C	43.97	7.89	369.10	138.87	
1D	64.76	423.16	4666.12	688.36	
2D	127.42	191.84	1279.32	225.92	9013.65
3D	9.00	51.29	1148.63	137.81	

Each cell corresponds to a methylation state pair. The row indicates the sample and initial methylation state. 1 corresponds to STL001 spleen, 2 to STL002 spleen, and 3 to STL003 spleen. The column indicates the terminal methylation state. Numerical values indicate the chi-squared contribution of a methylation state pair to the chi-squared test comparing all state pairs that begin with the same state (e.g., AA, AB, AC, AD). For example, the first number (11.07) is sample 1’s (STL001) AA pair’s contribution to the chi-squared test statistic (1323.14 in Table 3’s column A and Table 4’s last column)

In Fig. 3, the turquoise, dark green, and orange bars on the “by.sample” bar graphs display the primary contributions – how each methylation state pair contributes to the chi-squared statistic. The “by.sample” graphs can also indicate secondary contributions. That is, how much each individual sample contributes to the primary methylation state pair contributions. The blue, red, green and purple “by.methy.state” bar graphs display the primary contributions (per bar) – how each sample contributes to the entire chi-squared test statistic. The graphs also indicate secondary contributions within each bar. That is, how much each methylation state pair contributes to the primary sample contribution. The length of the bar or bar-segment corresponds to the amount of contribution to the overall chi-squared statistic by a specific sample or methylation state. Our analysis results show that for co-methylation patterns with methylation state pairs that start with B, C, and D, sample 1 or STL001 contributes the most (see the green or bright green bars in “B/C/D.contr.by.methy.state”). Thus, in regards to the co-methylation patterns of methylation state changes starting with B, C, and D, STL001 has the patterns that are the most different. However, for the co-methylation pattern of methylation state changes starting with A, sample 3 or STL003 is the most different one, see the last/fourth orange bar in the “A.contr.by.sample” of Fig. 3 top left panel.

Both the 3-sample chi-squared test result (i.e., small *p*-values in Table 3) and the above “contribution” Fig. 3 and Table 4 show that the three spleen samples have significantly different co-methylation patterns. These results seem to be contradictory with our intuitive findings shown in Fig. 2, that is, these 3 samples are not very different. This discrepancy, especially the very significant p-value may be caused by a large-count or large sample size effect, since chromosome 1 is very long, including 2,284,470 CG sites. Under the assumption that a subset of our chromosome is representative of the entire chromosome, we divide the analysis 1 count data (the left panel of Table 2) by 10s (by 1, 10, 100, and 1000). This helps to lessen the potential effect of a large sample size, as this “dividing” method creates a simulated 100, 10, 1%, and .1% of our data. For example, the STL001 AA count in Table 2 is originally 15,913, but after dividing by 10, it becomes 1591; After dividing by 100, it becomes 159.

Once we divide our method 1 data (the methylation state pair counts) by these factors of 10, we run the chi-squared test on the “modified” count data. We determine whether the *p*-values remain significant as the data pool gets smaller. If we see that the p-value becomes less significant as the sample size becomes smaller, we may conclude that the original p-values are not accurate due to a large sample size. The results will also tell us whether the methylation-change patterns between our three samples are significantly different. Our data for the chi-squared tests on all three spleen samples are shown in Table 5. We find that the test results become less significant as the sample size decreases. This shows that we do initially get a very small *p*-value because of the large sample size effect. We can determine that there is not a confirmation of a significant difference in the co-methylation patterns of three spleen samples. The patterns we observed in our Analysis 1 result may be applicable to other spleen samples, if confirmed by future studies with more normal spleen samples.

**Table 5:** Chi-squared test results after addressing the large-count issue (for 3S1T data)

Division	Value	A	B	C	D
1	*p*-value	1.06E-282	4.98E-225	1.80e-319	0
	Chi-square	1323.14	1056.65	1492.95	9013.66
	Degree of freedom	6	6	6	6
10	p-value	4.17E-26	1.64E-20	1.07E-29	1.92E-191
	Chi-square	132.32	105.67	149.33	901.34
	Degree of freedom	6	6	6	6
100	p-value	0.041	0.11	0.021	2.94E-17
	Chi-square	13.13	10.42	14.93	90.06
	Degree of freedom	6	6	6	6
1000	p-value	0.95	0.98	0.96	0.16
	Chi-square	1.64	1.19	1.48	9.3
	Degree freedom	6	6	6	6

The rows indicate the factor that the data are divided by. The second column indicates the type of value from the chi-squared test. The remaining columns indicate which co-methylation pattern the chi-squared test is used to compare

#### Analysis 2 results of 3S1T data

To analyze and compare how long SMRs are, we gather 6 summary values: the minimum, 1st Quartile, median, mean, 3rd Quartile, and maximum, for the number of CG sites (count) and distance in base pairs (length) for each SMR in each sample. Note, to avoid the impact of an extreme outlier, we remove the largest count/length and summarize our counts and lengths in Table 6. This table displays the distribution of count and length so that we can visualize and compare the distribution among our three spleen samples. Table 6 shows that the distributions of SMR length or CG-number count skew to the right. Most SMR are very short. The median SMR length is just about 100 to 120 base pairs for methylation state A and 300 to 450 base pairs for state D. The median count among SMRs is about 2 to 6 CG sites. Only a small proportion (< 25%) of the SMRs are longer than 1000 base pairs.

**Table 6:** SMR summaries of 3S1T data

	Count Summaries						Length Summaries					
STL001	Min.	1st Qu.	Median	Mean	3rd Qu.	Max.	Min.	1st Qu.	Median	Mean	3rd Qu.	Max.
A	2	2	4	10.72	13	152	2	35	97.5	188.5	231	3500
B	2	2	2	2.43	3	12	2	16	43	84.91	101	1512
C	2	2	2	2.43	3	13	2	27	68	132.1	161	4454
D	2	3	6	10.06	13	221	2	138	459	943.4	1190	50,660
STL002	Min.	1st Qu.	Median	Mean	3rd Qu.	Max.	Min.	1st Qu.	Median	Mean	3rd Qu.	Max.
A	2	2	4	11.1	13	193	2	36	103	200.5	250	3604
B	2	2	2	2.44	3	12	2	18	47	93.36	115	1951
C	2	2	2	2.46	3	13	2	32	83	157.9	198	3328
D	2	3	5	8.4	10	158	2	109	357	735.2	914	50,400
STL003	Min.	1st Qu.	Median	Mean	3rd Qu.	Max.	Min.	1st Qu.	Median	Mean	3rd Qu.	Max.
A	2	2	4	17.06	11	319	2	43	127	300.3	341	4760
B	2	2	2	2.55	3	17	2	21	52	100.4	119	2184
C	2	2	2	2.5	3	23	2	36	93	179.2	226	3419
D	2	3	5	8.97	10	252	2	116	369	755.4	925	50,420

The first column designates the type of SMR. The remaining columns are the summary. For example, for sample STL001, the “A” row is the summary of the “AA … A” type SMRs’ count and length, and the “B” row is the summary of the “BB … B” type SMRs’ count and length

The summary in Table 6 will help us to determine whether there are any significant differences among the three samples. Table 6 shows that the right-skew patterns of the A, B, and C methylation-state count vary among three spleen samples. We also notice that the SMRs of methylation state D are an exception to Cokus et al’s paper [11], where they observe a correlation between methylated cytosines for distances up to 5000 bases in Arabidopsis. The D regions can reach up to 50,000 bases in a human sample. Next, we investigate more on this question: Are these distribution differences significant? We answer this question using Kruskal Wallis tests.

#### Comparing the distribution of the count and length of SMRs across three spleen samples

We use the Kruskal Wallis test to analyze whether there is a significant difference between the distribution of count and length of the SMRs for each methylation state of each sample. The null (H_0_) and alternative (H_a_) hypotheses of the Kruskal Wallis test are listed below. H_0_: The CG count (or length) distribution of SMRs are the same among three samples. That is, there is no difference among the CG count distribution of the three samples. H_a_: There are differences for the SMR CG count distribution among the spleen tissue of three samples. We use our original count data, which are summarized in Table 6, as input for each sample to conduct the Kruskal Wallis test. The test results are shown in Table 7. As displayed in this table, most of the *p*-values are very small, except for two p-values in the STL001vSTL002 comparison. This finding means that the three spleen samples are significantly different regarding the distribution of SMR length and the distribution of SMR CG-site count.

**Table 7:** Kruskal Wallis results of 3S1T data

	All three spleen SMR CG count results				All three spleen SMR length results			
	A	B	C	D	A	B	C	D
p-values	7.99E-16	1.19E-11	2.94E-21	0	2.06E-74	1.83E-24	8.00E-212	0
x-squared	69.53	50.31	94.55	2965.34	339.34	109.32	972.14	2508.34
df	2	2	2	2	2	2	2	2
	STL001vSTL002 SMR CG count results				STL001vSTL002 SMR length results			
	A	B	C	D	A	B	C	D
*p*-values	0.02	0.38	6.04E-08	0	6.48E-04	7.77E-09	1.51E-78	0
x-squared	5.35	0.78	29.35	2459.83	11.63	33.33	352.05	2130.60
df	1	1	1	1	1	1	1	1
	STL002vSTL003 SMR CG count results				STL002vSTL003 SMR length results			
	A	B	C	D	A	B	C	D
*p*-values	6.33E-09	8.68E-09	2.25E-06	4.60E-03	5.57E-48	8.72E-07	1.79E-42	7.12E-08
x-squared	33.73	33.12	22.37	8.03	211.80	24.19	186.56	29.03
df	1	1	1	1	1	1	1	1
	STL001vSTL003 SMR CG count results				STL001vSTL003 SMR length results			
	A	B	C	D	A	B	C	D
*p*-values	8.29E-17	5.46E-10	3.34E-22	0	1.88E-68	1.20E-25	1.22E-212	0
x-squared	69.34	38.51	93.89	2040.71	305.71	109.59	968.57	1654.28
df	1	1	1	1	1	1	1	1

The rows indicate the Kruskal Wallis test results (p-values, chi-squared statistic, and degree of freedom). The columns indicate the co-methylation state (e.g., “A”) being compared by the test. The input files or datasets of Kruskal Wallis tests are the count and length data that are used to generate the summary in Table 6

### 1S8T data analysis results

After addressing whether different samples of the same tissue have significantly different co-methylation patterns using the 3S1T data, we next show the results of investigating if different tissues of the same sample have significantly different co-methylation patterns using the 1S8T data.

#### Analysis 1 result of 1S8T data

**Fig. 4:**
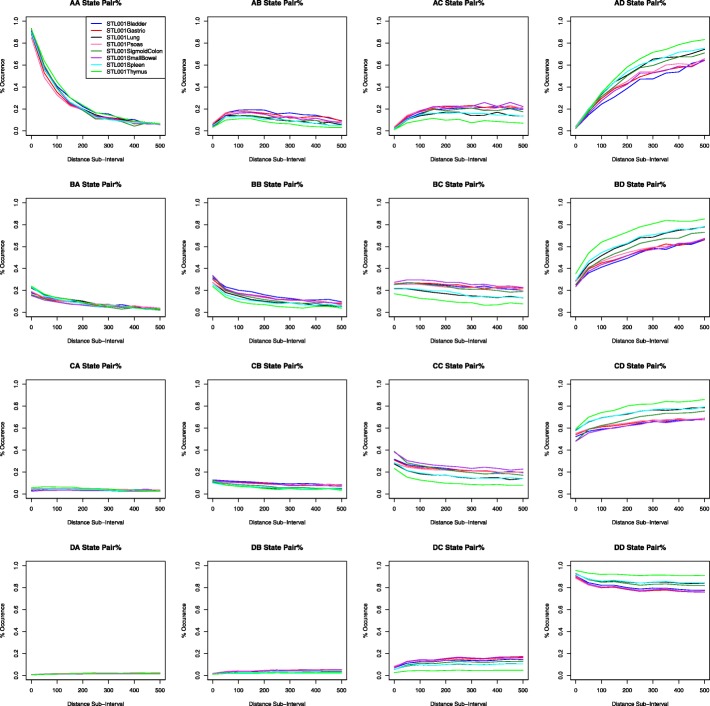
Comparing co-methylation patterns among eight different tissues (for 1S8T data). These graphs are created by plotting the percent occurrence for each distance sub-interval incremented by 50: [0, 50), [50, 100), [100, 150), and so on to [500, Inf). The x-axis indicates distance between CG sites. For example, a distance sub-interval of [50, 100) means that all paired CG sites are within 50 to 100 base pairs of each other. The y-axis indicates the percent occurrence of the methylation state pair as designated by the graph title. Different lines correspond to eight different tissues

We can analyze this dataset in two ways. First, we look at the overall theme for all eight tissues; we want to figure out which methylation state pair occurs most frequently. As shown in Table 8, for the bladder data, the AA methylation state pair has a percentage of 83.26% and the DD methylation state pair has a percentage of 86.54%. We notice that the different tissues display similar patterns; the AA and DD methylation state pairs have the highest percentage. We find that methylation tends to remain within the same methylation level if it is high or low. On the other hand, medium levels of methylation trend towards higher methylation states. This pattern continues throughout all eight tissues. However, there is some variation or difference among these eight tissues. We will investigate how closely these eight tissues follow the patterns we observed, so we graph percentage data for each tissue at certain distance intervals, see Fig. 4. This figure shows that there are some differences among those 8 tissues, especially for AD, BD, CD, and DD state pairs.

**Table 8:** Methylation state change of 1S8T data

	STL001 Bladder				STL001 Gastric			
	A	B	C	D	A	B	C	D
A	83.26	6.62	3.9	6.23	79.25	7.53	5.08	8.14
B	14.03	24.6	25.51	35.86	13.96	22.65	25.9	37.49
C	3.9	11.65	27.34	57.11	3.98	10.64	26.56	58.82
D	1.05	2.81	9.59	86.54	1.19	2.91	10.98	84.92
	STL001 Lung				STL001 Psoas			
	A	B	C	D	A	B	C	D
A	79.58	6.78	4.19	9.45	74.16	8.84	6.6	10.4
B	17.2	20.25	20.32	42.23	14.55	21.15	24.93	39.38
C	4.47	8.32	22.13	65.09	4.75	10.78	24.99	59.49
D	1.16	2.04	7.49	89.32	1.39	3.15	10.89	84.57
	STL001 SigmoidColon				STL001 SmallBowel			
	A	B	C	D	A	B	C	D
A	86.99	4.38	2.73	5.9	85.48	5.08	3.55	5.89
B	12.46	23.87	25.1	38.57	11.66	24.47	27.92	35.96
C	2.81	9.03	30.42	57.75	2.92	10.32	31.69	55.07
D	0.85	1.95	8.02	89.18	0.94	2.51	10.46	86.09
	STL001 Spleen				STL001 Thymus			
	A	B	C	D	A	B	C	D
A	81.37	6.16	3.83	8.64	86.27	4.12	2.19	7.42
B	17.04	18.69	20.9	43.37	18.33	17.02	14.77	49.89
C	4.03	8.18	22.85	64.95	5.66	8.92	17.17	68.24
D	1.07	1.94	7.5	89.49	1.01	1.52	3.49	93.98

The “A”, “B”, “C”, and “D” rows indicate the first methylation state of the CG pair. The “A”, “B”, “C”, and “D” columns indicate the second methylation state of the pair. The percentage in each cell is the count of each methylation state pair divided by the row sum of the counts. Note, the meaning or interpretation of this table is similar to Table 2, which is for the 3S1T data

Next, we conduct chi-squared tests to compare the 8 tissues, see the top panel of “divide by 1” in Table 9. When comparing all eight tissues together, we find that there are statistically significant differences in the co-methylation patterns among 8 tissues. To avoid the large-count issue, we divide the input data by factors of 10: 1, 10, 100, and 1000 as we did for the 3S1T data. The division of the data by 100 seems to help the results to be more accurate. As for the division by 1000, the count (or expected count for the chi-square test) is less than 5, so we will not use this result. Instead, we use the results with the division by 100, which still shows there are small *p*-values, that is, the 8 tissues have significantly different co-methylation patterns.

**Table 9:** Chi-squared test results divided by factors of 10 (for 8 tissues of 1S8T data)

Division	Value	A	B	C	D
1	*p*-value	0	0	0	0
	Chi-square	22,200.33	10,414.6	19,217.69	116,856.2
	Degree of freedom	21	21	21	21
10	*p*-value	0	4.15E-207	0	0
	Chi-square	2221.18	1041.43	1921.48	11,686.62
	Degree of freedom	21	21	21	21
100	*p*-value	1.35E-35	6.57E-13	1.07E-29	1.51E-234
	Chi-square	222.39	103.62	192.51	1170
	Degree of freedom	21	21	21	21
1000	*p*-value	0.42	0.96	0.58	3.16E-15
	Chi-square	21.64	10.95	19.11	116.47
	Degree of freedom	21	21	21	21

The rows indicate the factor that the data are divided by. The second column indicates the type of value from the chi-squared test. The remaining columns indicate which co-methylation pattern the chi-squared test is used to compare. Note, the meaning or interpretation of this table is similar to Table 5, which is for the 3S1T data

**Fig. 5:**
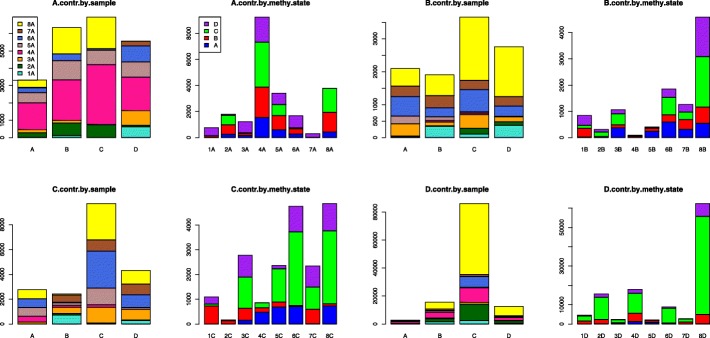
Comparing chi-squared test contributions by sample and by methylation state (for 1S8T data). The title indicates the co-methylation pattern compared by the chi-squared test. The x-axis reveals the second methylation state of the pair. For example, if the graph title contains “A”, then bar “B” displays the contribution of the specific state pair “AB” to the chi-squared value. The x-axis can also indicate sample number (i.e., “1C”, “2C”, …, “8C”). The number 1, 2, …, 8 corresponds to the 8 tissue, that is, 1: Bladder, 2: Gastric, 3: Lung, 4: Psoas, 5: Sigmoid Colon, 6: Small Bowel, 7: Spleen, and 8: Thymus. The y-axis displays the chi-squared contribution value

We also try to break the chi-squared test results down into the contributions that each methylation state and/or tissue contributes to the chi-squared value. We would like to know if any one tissue/methylation state has a bigger effect on the chi-squared test, and if so, whether we should remove that tissue/methylation state as an outlier. We display our Analysis 1 chi-squared contribution using bar graphs, see Fig. 5. In this figure, we can see that the 8th tissue, thymus, contributes most to the difference. We can also see that the 4th tissue, Psoas, contributes occasionally. We remove the 8th tissue, thymus, from our dataset, and re-run the chi-squared test on our data to see if the results are more accurate. Our results are displayed in Table 10. We can see that the *p*-values for this test remain zero, even though we remove a tissue. So, we try to divide the chi-squared input of the seven tissues by factors of 10, similar to what we have done above, and observe the chi-squared results. The result of dividing by 100 still have significant difference among 7 tissues; however, the results of dividing by 1000 only show a significant difference for D states, but not for A, B, or C states.

**Table 10:** Chi-squared test results of 7 tissues divided by factors of 10 (for 1S8T data)

Division	Value	A	B	C	D
1	*p*-value	0	0	0	0
	Chi-square	17,285.29	5441.81	14,025.19	41,241.11
	Degree of freedom	18	18	18	18
10	*p*-value	0	5.53E-104	5.10E-287	0
	Chi-square	1729.24	544.06	1402.08	4124.86
	Degree of freedom	18	18	18	18
100	*p*-value	2.50E-27	1.73E-05	4.86E-21	1.62E-76
	Chi-square	172.84	54.16	140.62	413.19
	Degree of freedom	18	18	18	18
1000	*p*-value	0.55	1	0.72	1.40E-03
	Chi-square	16.58	5.8	14.19	41.28
	Degree of freedom	18	18	18	18

The rows indicate the factor that the data are divided by. The second column indicates the type of value from the chi-squared test. The remaining columns indicate which co-methylation pattern the chi-squared test is used to compare. Note, the meaning or interpretation of this table is similar to Table 5 (for the 3S1T data) and Table 9 (for the 1S8T data with 8 tissues)

#### Analysis 2 result of 1S8T data

We would like to analyze SMRs. We first group CG sites that are similarly methylated and determine the number or count of CG sites in each SMR and the length (in base pairs) of each SMR. We then create summaries of the CG site number count and length of SMRs, see Table 11. Similar to Table 6 (summary for 3S1T data), Table 11 shows that the distributions of SMR length or CG-number count skew to the right. Most SMRs are very short. The median SMR length is around 100 to 130 base pairs for methylation state A and 300 to 500 base pairs for state D of all tissues except thymus. The median count among SMRs is about 2 to 6 CG sites. Next we determine whether SMRs of the same methylation level have different counts/lengths.

**Table 11:** SMR summaries of 1S8T data

	Bladder Count Summaries							Bladder Length Summaries					
	Min.	1st Qu.	Median	Mean	3rd Qu.	Max.		Min.	1st Qu.	Median	Mean	3rd Qu.	Max.
A	2	2	4	15.59	11	344	A	2	40	121	283.7	331	8693
B	2	2	2	2.54	3	16	B	2	24	62	117.9	148	2061
C	2	2	2	2.49	3	13	C	2	32	84	157	201	2882
D	2	3	5	9.03	11	213	D	2	118	390	789.4	1003	100,200
	Gastric Count Summaries							Gastric Length Summaries					
	Min.	1st Qu.	Median	Mean	3rd Qu.	Max.		Min.	1st Qu.	Median	Mean	3rd Qu.	Max.
A	2	2	4	10.66	12	170	A	2	34	95	193.7	239	5156
B	2	2	2	2.48	3	15	B	2	22	57	109.5	136	2534
C	2	2	2	2.46	3	15	C	2	31	82	585	202	21,050,000
D	2	3	5	7.74	10	146	D	2	102	335	665.6	853	50,630
	Lung Count Summaries							Lung Length Summaries					
	Min.	1st Qu.	Median	Mean	3rd Qu.	Max.		Min.	1st Qu.	Median	Mean	3rd Qu.	Max.
A	2	2	4	9.33	11	137	A	2	34	92	176.5	220	5765
B	2	2	2	2.44	3	11	B	2	18	45	87.52	106	1549
C	2	2	2	2.4	3	14	C	2	26	66	875	161	21,050,000
D	2	3	6	9.68	12	208	D	2	135	447	908.6	1162	100,200
	Psoas Count Summaries							Psoas Length Summaries					
	Min.	1st Qu.	Median	Mean	3rd Qu.	Max.		Min.	1st Qu.	Median	Mean	3rd Qu.	Max.
A	2	2	4	8.23	9	132	A	2	30	83	157.7	193	4512
B	2	2	2	2.42	3	12	B	2	21	54	106.1	128.8	2690
C	2	2	2	2.41	3	12	C	2	29	77	152.1	189	3643
D	2	3	5	7.42	9	169	D	2	95	315	652.1	824	100,300
	SigmoidColon Count Summaries							SigmoidColon Length Summaries					
	Min.	1st Qu.	Median	Mean	3rd Qu.	Max.		Min.	1st Qu.	Median	Mean	3rd Qu.	Max.
A	2	2	4	22.19	20	374	A	2	42	136	351.7	428	5156
B	2	2	2	2.64	3	25	B	2	20	52	96.21	121	1835
C	2	2	2	2.69	3	48	C	2	34	86	678	201	21,050,000
D	2	3	6	10.65	13	294	D	2	150	488.5	963.3	1227	100,200
	SmallBowel Count Summaries							SmallBowel Length Summaries					
	Min.	1st Qu.	Median	Mean	3rd Qu.	Max.		Min.	1st Qu.	Median	Mean	3rd Qu.	Max.
A	2	2	4	20.62	17	390	A	2	42	132	340.6	397	8889
B	2	2	2	2.62	3	38	B	2	23	59	108.5	135	1714
C	2	2	2	2.66	3	34	C	2	37	95	560	226	21,050,000
D	2	3	5	8.68	10	233	D	2	117	375	735.5	940	50,460
	Spleen Count Summaries							Spleen Length Summaries					
	Min.	1st Qu.	Median	Mean	3rd Qu.	Max.		Min.	1st Qu.	Median	Mean	3rd Qu.	Max.
A	2	2	4	10.73	13	164	A	2	35	98	188.8	231	4941
B	2	2	2	2.43	3	14	B	2	16	43	85.06	101.8	1534
C	2	2	2	2.43	3	14	C	2	27	68	851	161	21,050,000
D	2	3	6	10.06	13	265	D	2	138	459	944	1190	100,200
	Thymus Count Summaries							Thymus Length Summaries					
	Min.	1st Qu.	Median	Mean	3rd Qu.	Max.		Min.	1st Qu.	Median	Mean	3rd Qu.	Max.
A	2	2	4	17.3	14	346	A	2	48	137	302.3	360	4927
B	2	2	2	2.44	3	23	B	2	14	36	72.25	86	1443
C	2	2	2	2.36	2	20	C	2	20	51	102.1	118	2152
D	2	4	9	17.04	21	408	D	2	229	818	1696	2120	100,200

The first column designates the type of SMR. The remaining columns are the summary. For example, for the bladder tissue, the “A” row (i.e., the 3rd row) is the summary of the “AA … A” type SMR’s count and length, and the “B” row (4th row) is the summary of the “BB … B” type SMR’s count and length of this tissue

We run the Kruskal Wallis test on all eight tissues to determine if the distribution of counts and distances of each type of SMR are significantly different. Our initial results are displayed below in Table 12. As we can see, the *p*-values for the count in columns C and D, and the p-values for distance in columns A, C, and D are all nearly zero. The remaining p-values are also extremely small. This shows that those 8 tissues are significantly different.

**Table 12:** Kruskal Wallis test results of 1S8T data

	SMR CG Site Count				SMR Length			
	A	B	C	D	A	B	C	D
*p*-values	6.73E-97	3.13E-91	0	0	0	1.79E-260	0	0
x-squared	467.78	441.39	2645.20	32,987.79	2168.20	1225.88	3020.88	29,507.40
df	7	7	7	7	7	7	7	7

The rows indicate the Kruskal Wallis test results (p-values, chi-squared statistic, and degree of freedom). The columns indicate the co-methylation state (e.g., “A”) being compared by the test. The input files or datasets of Kruskal Wallis tests are the count and length data that are used to generate the summary in Table 11. For example, the second column (for methylation state A or for “AA … A” type SMR) is the test result of using all the counts of “AA … A” SMRs that are used to generate the summary in the “A” rows for each of the 8 tissues in Table 11

In summary, in the Result section, we have shown the results of analyzing two datasets, 3S1T and 1S8T, to address a few questions mentioned in the Introduction section.

## Discussion

Our research work is a specific study with a focus on the analysis of within-sample co-methylation patterns in normal DNA samples. The understanding of this specific type of methylation patterns may provide helpful information and insights on other methylation studies. These studies may include the analysis of methylation patterns for specific genes or regions (e.g., long non-coding RNAs [16])*,* identifying differential methylation [24–27]*,* classifying large methylation data [28–30]*,* integrating methylation with other data (e.g., gene expression data) [31–33], and analyzing pan-cancer DNA methylation [20, 34–37].

To the best of our knowledge, there is not much research work done on the WS co-methylation patterns of normal DNA samples, although there are a larger number of papers on BS co-methylation. Thus, in this paper, we conduct some preliminary analyses on WS co-methylation for normal samples. However, our research work has certain limitations. First, this study is only conducted on normal samples, but this type of within-sample analysis can be performed on other datasets, for example, cancer methylation data with multiple samples. In fact, we have submitted a paper on comparing the within-sample co-methylation between normal and cancer samples. In that paper, we find that the breast cancer sample’s co-methylation pattern is very different from the patterns reported in this manuscript. But the breast normal sample has a similar pattern as we found in the 1S8T data of the current study. Therefore, co-methylation patterns of other normal samples or other datasets could be very similar to what we showed as we used a relatively large number of different normal tissues in this paper. The second limitation is that our study is only based on basic statistical analysis. More complex probability models (e.g., Hidden Markov Models) and methods (e.g., Bayesian methods or machine learning) may be used to further investigate within-sample co-methylation. Developing a new method based these ideas and methods is beyond the scope of this paper. However, we do plan to implement these models and ideas in other projects in the near future. The third limitation is that we did not investigate the relationship between WS co-methylation and the genomic context and/or the topology of CG sites [38]. It would be biologically meaningful to investigate in detail how WS co-methylation patterns are related to the genomic compartments, e.g., gene body, gene promoter, and CpG islands. To make it easier for other readers to interpret the co-methylation CG sites or patterns, we provide an annotation code (*annotation.R*) in the Additional File 1 that can report the location of identified CG sites, that is, which gene body or promoter it belongs to. Note, the Additional File 1 includes all R scripts we wrote for the analysis of this paper. In addition, it is also useful to study how these patterns are related to the density of CG sites (or CG clusters) in an entire genome and study how CG sites collaborate to maintain co-methylation patterns [38, 39]. These are all great aspects to investigate further in future research studies and we do plan to conduct research on these topics.

In order to confirm the validity of our findings, we have also conducted our analysis on the same tissues and samples from chromosome 2. We find similar patterns and can make the same conclusion. In order to avoid redundancy, we do not show the results of chromosome 2 data analysis and only show the results of analyzing chromosome 1 in this paper.

## Conclusion

In this paper, we have conducted analyses to study the within-sample co-methylation patterns in normal DNA samples. We have investigated if the co-methylation patterns of the same tissue across several samples are different and if the co-methylation patterns of various tissues of the same sample are different. We have used two analyses methods to analyze two datasets, 3S1T and 1S8T. Based on the 3S1T data, we find there is not significant co-methylation difference among the same spleen tissues of three different samples. However, the analysis results of 1S8T data show that there were significant differences among eight tissues of one sample. For both 3S1T and 1S8T data, we find that the no/low methylation state A and high/full methylation state D tend to remain the same along a chromosome region. We also find that the low/partial methylation state B and partial/high methylation state C tend to change to higher methylation states along a chromosome. Furthermore, we find that the distribution of SMR length is skewed to the right and most SMRs are very short (with only a few hundred base pairs) and only a small proportion of SMRs are longer than 1000 base pairs. In this paper, we have addressed a few questions regarding within-sample co-methylation. Our answers and analysis results may help researchers to have a deep understanding of co-methylation patterns and thus to improve DNA methylation assays and statistical analyses for other methylation studies.

## Additional file

Additional file 1: All R scripts for the analysis of this paper. (ZIP 20 kb)

## Abbreviations

CG: Cytosine-guanine
SMR: Similarly methylated region
WGBS: Whole genome bisulfite sequencing
